# Associations Between Sociodemographic Characteristics and Perceptions of the Built Environment With the Frequency, Type, and Duration of Physical Activity Among Trail Users

**Published:** 2012-02-09

**Authors:** Andréa L. Maslow, Julian A. Reed, Anna E. Price, Steven P Hooker

**Affiliations:** R. Stuart Dickson Institute for Health Studies, Carolinas HealthCare System; Department of Exercise Science, Sacred Heart University, Fairfield, Connecticut; Department of Exercise Science, Sacred Heart University, Fairfield, Connecticut; Department of Exercise Science, Arnold School of Public Health, University of South Carolina and Prevention Research Center, Arnold School of Public Health, University of South Carolina, Columbia, South Carolina

## Abstract

**Introduction:**

Rail trails are elements of the built environment that support the Task Force on Community Preventive Services' recommendation to create, or enhance access to, places for physical activity (PA). The purpose of this study was to examine the associations between sociodemographic characteristics and perceptions of the built environment with the frequency, type, and duration of PA among users of an urban, paved rail trail segment.

**Methods:**

Interviewers conducted intercept surveys with 431 rail trail users and analyzed data by using logistic regression to estimate odds ratios between sociodemographic characteristics and perceptions of the built environment on the frequency, type, and duration of PA performed on the trail.

**Results:**

Adults who used the trail in the cool months, traveled to the trail by a motorized vehicle, used the trail with others, and had some graduate school education visited the trail less often. Younger adults, men, whites, and those with some graduate school education were more likely to engage in vigorous activities on the trail. Adults who traveled to the trail by a motorized vehicle spent more time engaged in PA on the trail.

**Conclusion:**

Our results suggest that the most frequent users of a rail trail for PA are those who use the trail alone and travel to the trail by bicycle or on foot. Trails are an aspect of the built environment that supports active lifestyles, and future studies should evaluate different types of trails among more diverse populations and locations.

## Introduction

The US Department of Health and Human Services recommends that adults engage in at least 150 minutes per week of moderate-intensity aerobic physical activity (PA), 75 minutes per week of vigorous-intensity aerobic PA, or an equivalent combination of moderate- and vigorous-intensity aerobic PA (1). However, sedentary behavior and lack of PA continues to be a public health issue among adults (2-4). Ecological approaches that focus on the combined effect of public policy, social systems, and physical environments have been emphasized as a means of modifying PA at the population level (5-7). The US Task Force on Community Preventive Services recommends the creation of or enhanced access to places for PA combined with informational outreach activities as an effective method for influencing people to exercise more (8). However, this recommendation was based largely on 6 worksite interventions with limited evidence from studies designed to modify built environmental features of a community. Therefore, although the creation of community rail trails, former railways converted into multi-use trails, is an element of the built environment that supports this recommendation (9-14), more research on the association between rail trails and PA is needed.

There are positive associations between trail use and frequency (9) and duration of PA (9,15) among residents in the surrounding communities, especially among novice exercisers (16). Research also suggests that people who use trails are more likely to meet national PA guidelines than those who rarely or never use trails (17). Several studies that have examined demographic characteristics of trail users have indicated that most are younger adults (14,15), more educated (11,15), and white (14,18), but the association between trail use and sex is inconsistent (12,14,15,17). Proximity to exercise facilities may be a potential predictor of PA behavior (19,20); although most trail studies examining this relationship have indicated a positive association between proximity and trail use (15), some suggest proximity may not be a significant factor (9,14).

Evaluating the factors that affect trail use among community residents is critical to public health planning, especially in the South, which has some of the highest rates of physical inactivity (21,22) and chronic disease (23). However, most of the aforementioned trail studies have evaluated trail use as the number of people using the trail at a given time and did not assess frequency, type, and duration of PA on the trail. We address research gaps by examining the associations between sociodemographic characteristics and perceptions of the built environment with the frequency, type, and duration of PA on a newly converted urban rail trail.

## Methods

### Study setting and population

This study was conducted from 2006 through 2009 with adults aged 18 years or older who were using a 2-mile, paved, urban rail trail in Spartanburg, South Carolina. In 2010, approximately 39,000 people resided in Spartanburg, of whom 56% were female, 47% were white, and 50% were black. Of residents at least aged 25 years, 72% had a high school diploma and 26% had a bachelor's degree (24). The rail trail used in this study was funded in 2005 and parallels 2 major city streets and 2 diverse residential neighborhoods.

### Trail intercept survey

Intercept surveys are in-person, on-site interviews that are used when respondents cannot easily be reached at a later time.Troped et al (18) developed the brief, 17-item trail intercept survey with test-retest reliability ranging from K = 0.65 to 0.96 for categorical items and *r* = 0.62 to 0.93 for continuous items.

Data collection procedures are presented elsewhere (18). Briefly, trained interviewers positioned themselves at target areas on the trail and asked adult trail users passing the area if they were willing to participate in a survey. Interviewers confirmed that trail users were at least aged 18 before conducting the survey and informed trail users of their rights as research participants before administering the survey. Once the survey was completed, interviewers approached the next observed trail user to conduct an interview. Each year, interviewers attempted to intercept trail users at 4 times of the day for 7 consecutive days during each of the 4 seasons, to capture a representative sample and variations in seasons and times of use during the day and week. For approximately every 10 trail users observed on the trail, 1 interview was completed. Interviewers obtained verbal informed consent from all research participants. The University of South Carolina institutional review board approved the study procedures.

Sociodemographics obtained from the survey were age in years (18-39, 40-49, 50-59, or ≥60), sex, race (white or nonwhite) and educational attainment (high school graduate or less, some college, college graduate, or some graduate school or more). Perceptions of the built environment obtained were perceived maintenance of the trail (excellent or good to fair) and perceived safety and security of the trail (excellent or good to fair)*.* Other information and self-reported characteristics obtained from the survey were seasonality (cool months [October-March] or warm months [April-September]), proximity of the trail to the user's home or work in minutes (<15 or ≥15), transportation mode to the trail (bicycle/on foot or by motorized vehicle), and whether participants used the trail alone or with others. The main trail use outcomes were frequency of using the trail for PA (<5 d/wk or ≥5 d/wk), type of PA on the trail (walk or jog, run, bike, or skate), and duration of PA on the trail per visit in minutes (<45 or ≥45).

We categorized responses for all of these variables, except for age and frequency of PA, in the survey. The aforementioned categories for these variables were either created or collapsed as logically as possible to preserve sample sizes. For perceptions of the maintenance and safety and security of the trail, the "poor" category was removed for ease of interpretation and because there were so few of these responses; however, the results did not differ if we removed these responses from the good-fair category. We excluded first-time trail users (n = 40) because the frequency, type, and duration of PA questions were not applicable to this group.

### Statistical analysis

We used SAS version 9.2 (SAS Institute, Inc, Cary, North Carolina) to perform all analyses and computed descriptive statistics for all characteristics. First, we evaluated the bivariate associations between each characteristic and each trail use outcome by using logistic regression to estimate the odds ratios (ORs) and 95% confidence intervals (CIs). Second, for parsimonious models, only those characteristics significantly associated with the specific trail use outcome in the bivariate associations were controlled for in the adjusted analysis examining the associations on the frequency, type, and duration of PA on the trail. Third, we performed subsequent logistic regression analyses that examined the associations between sociodemographic characteristics and seasonality (independent variables) on the other self-reported characteristics (dependent variables). To illustrate the percentage of variation in the model explained by the independent variable(s), *R* ^2^ values were reported for all models. Only those trail users with complete data in each model were used in each analysis. All *P* values are 2-sided (α = .05).

## Results

At least half of the trail users interviewed were aged 50 or older, female, and white (Table 1). The demographic characteristics of this sample reflect those of more than 5,000 rail trail users observed during the same period (14). The likelihood of using the trail 5 or more days of the past week for PA was lower among trail users with some postgraduate education, compared with those with a high school degree or less (*P* = .003), and among those who used the trail with others, compared with those who used the trail alone (*P* = .004) (Table 2). The likelihood of using the trail 5 or more days of the past week for PA was higher among people who used the trail during warm months, compared with those who used the trail during cool months (*P* = .038), and among people who traveled to the trail by bicycle or on foot, compared with those who traveled to the trail by motorized vehicle (*P* = .006). Age, sex, race, proximity to the trail, and perceptions of the built environment were not significantly associated with frequency of trail use for PA and were not included in the adjusted, parsimonious models.

The likelihood of engaging in more vigorous activities (ie, jogging, running, biking, or skating) was lower among trail users aged 60 years or older, compared with those aged 18 to 39 years (*P* = .009); nonwhite trail users, compared with white trail users (*P* = .001); and people who used the trail with others, compared with those who used the trail alone (*P* = .004) (Table 2). The likelihood of engaging in more vigorous activities was higher among men than women (*P *< .001) and among trail users with more education compared with trail users with a high school degree or less. Seasonality, proximity to the trail, transportation to the trail, and perceptions of the built environment were not significantly associated with type of PA on the trail and were not included in the adjusted, parsimonious models. Trail users who traveled to the trail by bicycle or on foot were less likely to spend 45 minutes or more on the trail during PA (*P* = .004). No other characteristic was significantly associated with duration of PA on the trail; therefore, the final model evaluating duration of PA includes transportation to the trail as the only independent variable.

After controlling for education, seasonality, transportation to the trail, and using the trail with others, the adjusted associations of frequency of PA on the trail with education and seasonality intensified, while the adjusted associations of frequency of PA with transportation to the trail and using the trail with others were attenuated, yet remained significant (Table 3). After adjusting for age, sex, race, education, and using the trail with others, the adjusted associations on type of PA with age, sex, and using the trails with others intensified, the adjusted association with race remained the same, and the adjusted association with education was slightly attenuated, yet remained significant.

Men were more likely than women to travel to the trail by bicycle or on foot, and respondents with some college education were significantly less likely to travel to the trail by bicycle or on foot compared with respondents with a high school degree or less (Table 4). Men were significantly less likely than women to use the trail with others. Nonwhite trail users were less likely than white trail users to perceive the safety and security of the trail as excellent; trail users who used the trail during the warm months were significantly more likely than users of the trail during cool months to perceive the safety and security of the trail as excellent (*P* = .004) . Sociodemographic variables and seasonality were not significantly associated with proximity from home or work to the trail or perceived maintenance of the trail (data not shown).

## Discussion

We found that survey respondents who were most likely to use an urban rail trail in South Carolina for PA on 5 or more days per week had lower levels of education, traveled to the trail by bicycle or on foot, used the trail during the warm months, and used the trail alone. Respondents most likely to report walking as their primary activity on the trail were aged 40 years or older, female, and nonwhite; had a high school degree or less; and used the trail with others. Finally, respondents who traveled to the trail by a motorized vehicle were more likely to report spending 45 minutes or more on the trail for PA, and proximity to the trail was not significantly associated with either frequency, type, or duration of PA on the trail.

The age-related results we report, which show that younger adults were more likely to engage in more vigorous physical activities compared with older adults who prefer to walk, are comparable to those of other studies (25,26). The finding that men were more likely than women to travel to the trail by bicycle or on foot may be because men used the trail as a single section in a longer continuous run or bicycle ride; women may have reported traveling to the trail by a motorized vehicle because they use the trail for their entire bout of leisure-time PA. Women were more likely than men to use the trail with others. Wilcox et al also found that a larger proportion of women preferred to exercise with others compared with men (27). Women may be more likely to walk with others because being with other people may increase their perception of safety.

Our finding that trail users with a high school degree or less were more likely to frequent the trail 5 or more days per week for PA compared with trails users with some graduate school education or more conflicts with research that supports a positive relationship between education and trail use (11,15). One reason for this finding may be that trails are a more affordable option for PA for lower-income people than paying for a gymnasium membership or other formal exercise program. Our finding that trail users with a less formal education were more likely to walk on the trail may be because these users were walking for transportation rather than recreational purposes.

Proximity to PA resources has been identified as a possible correlate of PA (19,20). However, our results were similar to 2 studies (9,14) that did not find a significant association between proximity to a trail and PA behavior on the trail. If a trail is perceived as a valuable community asset, people likely will be willing to travel a reasonable distance to access the trail. A variety of adults used the trail year-round for multiple purposes, indicating that community trails can have wide appeal. However, promotional efforts should still target those who live or work closest to the trail.

Our study has limitations. The use of a self-reported PA measure is known to be affected by recall and social desirability bias. However, most trail research has used self-report surveys (18), primarily because of their low cost and ease of administration. Our study was cross-sectional and conducted in a southeastern city on 1 short rail trail segment; therefore, our results have limited generalizability. However, evaluating modifications to the built environment is needed to help support the idea that these modifications are worth the investment and can benefit communities in providing varied options for PA. Although staff incorporated multiple strategies in an attempt to interview a variety of users, they had difficulty persuading people who were running and bicycling on the trail to stop. Therefore, interviews were conducted mostly with adults who were walking on the trail. Ethnicity could not be examined successfully, because only 6 trail users identified themselves as Hispanic; no information was collected about income and car ownership. Including an overall PA outcome (ie, minutes/week) in the statistical models would have been preferable, but the survey asked trail users to report their frequency of PA as a continuous variable and their duration of PA as a categorical variable; therefore, creating a summarized PA variable was not feasible.

Another limitation was that whether trail users reported proximity to the trail from home or work by car, by bicycle, or on foot is unknown. However, we believe that the reported proximities do not represent different distances because most of the trail users likely reported traveling to the trail by car, and people typically choose to travel by car for transportation purposes if the walking time is more than 10 minutes (28). Therefore, a trail user who reported proximity in travel time to the trail by foot would likely be categorized the same if they had reported proximity in travel time by car of less than 15 minutes. Furthermore, proximity was asked as travel time to the trail because people are more likely to accurately recall time compared with determining distance. Finally, a few logistic regressions resulted in wide CIs, particularly with respect to the association between education and type of PA. These CIs were likely due to small cell sample sizes, which may have also resulted in limited power to detect differences in the other education categories.

Despite these limitations, the use of a reliable survey with sound psychometric elements enabled the collection of more detailed information on trail users than that provided by direct observation methods. Furthermore, data were collected during varied seasons, days of the week, and times of day to strengthen the representativeness of the findings. This study adds to the literature a better understanding of who uses trails for PA and how perceptions of the built environment, seasonality, and other self-reported characteristics are associated with frequency, type, and duration of PA on a rail trail. This information can reinforce the idea that trails are an aspect of the built environment that support active lifestyles and may be useful in developing more effective community-based interventions to promote trail use. Future studies should evaluate different types of trails among larger, more diverse populations and locations.

## Figures and Tables

**Table 1.** Survey Response Characteristics of 431 Adult Users of a Rail Trail in Spartanburg, South Carolina, 2006-2009

**Characteristic**	n (%)
**Age, y**	
18-39	103 (23.9)
40-49	97 (22.5)
50-59	101 (23.4)
≥60	113 (26.2)
Missing	17 (3.9)
**Sex**	
Female	229 (53.1)
Male	199 (46.2)
Missing	3 (0.7)
**Race**	
White	315 (73.1)
Nonwhite	105 (24.4)
Missing	11 (2.6)
**Educational attainment**	
≤High school graduate	79 (18.3)
Some college	90 (20.9)
College graduate	163 (37.8)
≥Some graduate school	97 (22.5)
Missing	2 (0.5)
**Seasonality**	
Cool (October-March)	240 (55.7)
Warm (April-September)	189 (43.9)
Missing	2 (0.5)
**Proximity from home or work to trail, min**	
<15	337 (78.2)
≥15	62 (14.4)
Missing	32 (7.4)
**Transportation mode to trail**	
Bicycle or on foot	105 (24.4)
Motorized vehicle	321 (74.5)
Missing	5 (1.2)
**On the trail . . .**	
Alone	187 (43.4)
With others	232 (53.8)
Missing	12 (2.8)
**Perceived maintenance of trail**	
Excellent	216 (50.1)
Good to fair	206 (47.8)
Missing	9 (2.1)
**Perceived safety and security of trail**	
Excellent	130 (30.2)
Good to fair	276 (64.0)
Missing	25 (5.8)
**No. of d/wk using trail for physical activity**	
0-4	314 (72.9)
5-7	102 (23.7)
Missing	15 (3.5)
**Type of physical activity on trail**	
Walk	313 (72.6)
Jog/run, bike, or skate	89 (20.6)
Missing	29 (6.7)^a^
**Time spent on trail during physical activity, min**	
<45	109 (25.3)
≥45	311 (72.2)
Missing	11 (2.6)

a Missing includes horse-back riding and "other" types of physical activity (n = 15).

**Table 2.** Unadjusted Associations Between Sociodemographic Characterisitcs, Seasonality, Perceptions of the Built Environment, and Other Self-Reported Characteristics With the Frequency, Type, and Duration of Physical Activity Among 431 Adult Users of a Rail Trail Conversion in Spartanburg, South Carolina, 2006-2009

Characteristic	≥5 d/wk PA on Trail^a^		Jog/Run/Bicycle on Trail^b^		≥45 min/Visit PA on Trail^c^	

	OR (95% CI)	*R* ^2^	OR (95% CI)	*R* ^2^	OR (95% CI)	*R* ^2^
**Age, y**						
18-39	1 [Reference]	0.013	1 [Reference]	0.026	1 [Reference]	0.012
40-49	1.65 (0.80-3.38)		0.54 (0.25-1.15)		1.59 (0.78-3.27)	
50-59	0.81 (0.37-1.78)		0.75 (0.36-1.55)		2.00 (0.94-4.23)	
≥60	1.33 (0.65-2.73)		0.33 (0.14-0.76)		1.18 (0.61-2.31)	
**Sex**						
Female	1 [Reference]	0.002	1 [Reference]	0.052	1 [Reference]	0.003
Male	1.23 (0.74-2.04)		3.22 (1.80-5.76)		0.76 (0.46-1.27)	
**Race**						
White	1 [Reference]	0.004	1 [Reference]	0.02	1 [Reference]	0.006
Nonwhite	1.39 (0.80-2.43)		0.38 (0.19-0.80)		1.54 (0.84-2.81)	
**Education**						
≤High school graduate	1 [Reference]	0.029	1 [Reference]	0.04	1 [Reference]	0.011
Some college	0.51 (0.24-1.11)		2.16 (0.70-6.62)		1.23 (0.53-2.99)	
College graduate	0.60 (0.31-1.15)		3.23 (1.17-8.87)		0.94 (0.46-1.94)	
≥Some graduate school	0.27 (0.11-0.65)		5.25 (1.83-15.04)		0.61 (0.28-1.33)	
**Seasonality**						
Cool (Oct-Mar)	1 [Reference]	0.015	1 [Reference]	0.011	1 [Reference]	0.006
Warm (Apr-Sep)	1.78 (1.07-2.98)		0.58 (0.33-1.03)		1.45 (0.86-2.45)	
**Proximity from home or work to trail, min**						
≥15	1 [Reference]	0.001	1 [Reference]	0.005	1 [Reference]	0.001
<15	0.84 (0.43-1.65)		0.51 (0.43-1.39)		0.79 (0.38-1.61)	
**Transportation mode to trail**						
Motorized vehicle	1 [Reference]	0.022	1 [Reference]	0.011	1 [Reference]	0.025
Bicycle or on foot	2.22 (1.26-3.93)		1.79 (0.97-3.31)		0.43 (0.25-0.76)	
**On the trail . . .**						
Alone	1 [Reference]	0.026	1 [Reference]	0.027	1 [Reference]	0.009
With others	0.47 (0.28-0.78)		0.44 (0.25-0.77)		1.58 (0.95-2.62)	
**Perceived maintenance of trail**						
Fair to good	1 [Reference]	0.003	1 [Reference]	0.006	1 [Reference]	.0001
Excellent	0.79 (0.47-1.31)		0.69 (0.40-1.19)		0.95 (0.57-1.59)	
**Perceived safety and security of trail**						
Fair to good	1 [Reference]	0.009	1 [Reference]	0.001	1 [Reference]	.0002
Excellent	1.59 (0.93-2.69)		0.82 (0.45-1.50)		0.93 (0.54-1.60)	

Abbreviations: PA, physical activity; OR, odds ratio; CI, confidence interval.

a Reference group is <5 d/wk of PA on trail.

b Reference group is walk on trail.

c Reference group is <45 min PA per visit on trail.

**Table 3.** Adjusted, Parsimonious Associations Between Sociodemographic Characteristics, Seasonality, and Other Self-Reported Characteristics With the Frequency and Type of Physical Activity Among 431 Adult Users of a Rail Trail Conversion in Spartanburg, South Carolina, 2006-2009

**Characteristic**	≥5 d/wk PA on Trail,^a^ OR (95% CI)	Jog/Run/Bicycle/Skate on Trail,^b^ OR (95% CI)
**Age, y**		
18-39	NA	1 [Reference]
40-49	NA	0.35 (0.15-0.81)
50-59	NA	0.41 (0.18-0.94)
≥60	NA	0.17 (0.07-0.44)
**Sex**		
Female	NA	1 [Reference]
Male	NA	3.31 (1.73-6.33)
**Race**		
White	NA	1 [Reference]
Nonwhite	NA	0.37 (0.16-0.87)
**Education**		
≤High school graduate	1 [Reference]	1 [Reference]
Some college	0.58 (0.26-1.32)	1.69 (0.50-5.71)
College graduate	0.62 (0.31-1.24)	2.32 (0.76-7.05)
≥Some graduate school	0.25 (0.10-0.63)	4.68 (1.42-15.45)
**Seasonality**		
Cool (Oct-Mar)	1 [Reference]	NA
Warm (Apr-Sep)	1.84 (1.07-3.15)	NA
**Transportation mode to trail**		
Motorized vehicle	1 [Reference]	NA
Bicycle or on foot	2.00 (1.08-3.69)	NA
**On the trail . . .**		
Alone	1 [Reference]	1 [Reference]
With others	0.51 (0.30-0.88)	0.49 (0.26-0.92)

Abbreviations: PA, physical activity; OR, odds ratio; CI, confidence interval; NA, not assessed.

a Reference group is <5 d/wk PA on trail. Adjusted *R* ^2^ for the full model = 0.12.

b Reference group is walk on trail. Adjusted *R* ^2^ for the full model = 0.24.

**Table 4.** Adjusted Associations Between Sociodemographic Characteristics and Seasonality and Other Self-Reported Characteristics With Perceptions of the Built Environment Among 431 Adult Users of a Rail Trail Conversion In Spartanburg, South Carolina, 2006-2009

**Characteristic**	Travel to Trail by Bicycle or on Foot,^a^ OR (95% CI)	On the Trail With Others,^b^ OR (95% CI)	Perceived Excellent Safety and Security of Trail,^c^ OR (95% CI)
**Age, y**			
18-39	1 [Reference]		
40-49	0.60 (0.31-1.18)	0.72 (0.40-1.30)	0.79 (0.41-1.53)
50-59	0.59 (0.30-1.15)	1.03 (0.56-1.89)	0.63 (0.33-1.22)
≥60	0.76 (0.40-1.44)	0.97 (0.54-1.74)	1.20 (0.65-2.24)
**Sex**			
Female	1 [Reference]		
Male	1.93 (1.21-3.08)	0.35 (0.23-0.53)	1.22 (0.78-1.91)
**Race**			
White	1 [Reference]		
Nonwhite	0.75 (0.41-1.40)	0.95 (0.55-1.64)	0.46 (0.25-0.86)
**Education**			
≤High school graduate	1 [Reference]		
Some college	0.43 (0.20-0.96)	1.25 (0.62-2.52)	1.39 (0.64-3.02)
College graduate	0.51 (0.25-1.02)	1.12 (0.59-2.12)	1.23 (0.59-2.53)
≥Some graduate school	0.78 (0.36-1.68)	1.15 (0.56-2.38)	1.43 (0.65-3.16)
**Seasonality**			
Cool (Oct-Mar)	1 [Reference]		
Warm (Apr-Sep)	1.24 (0.78-1.99)	1.22 (0.80-1.86)	1.95 (1.24-3.06)

Abbreviations: OR, odds ratio; CI, confidence interval.

a Reference group is travel to trail by motorized vehicle. Adjusted *R* ^2^ for the full model = 0.06.

b Reference group is on the trail alone. Adjusted *R* ^2^ for the full model = 0.10.

c Reference group is good to fair. Adjusted *R* ^2^ for the full model = 0.08.
